# Host microbiota can facilitate pathogen infection

**DOI:** 10.1371/journal.ppat.1009514

**Published:** 2021-05-13

**Authors:** Emily J. Stevens, Kieran A. Bates, Kayla C. King

**Affiliations:** Department of Zoology, University of Oxford, Oxford, United Kingdom; Geisel School of Medicine at Dartmouth, UNITED STATES

## Abstract

Animals live in symbiosis with numerous microbe species. While some can protect hosts from infection and benefit host health, components of the microbiota or changes to the microbial landscape have the potential to facilitate infections and worsen disease severity. Pathogens and pathobionts can exploit microbiota metabolites, or can take advantage of a depletion in host defences and changing conditions within a host, to cause opportunistic infection. The microbiota might also favour a more virulent evolutionary trajectory for invading pathogens. In this review, we consider the ways in which a host microbiota contributes to infectious disease throughout the host’s life and potentially across evolutionary time. We further discuss the implications of these negative outcomes for microbiota manipulation and engineering in disease management.

## Introduction

An infection by pathogens (and parasites) can vary from relatively benign to lethal. The degree of harm caused during infection can be driven by aspects of pathogen biology, such as transmissibility [1], infective dose [2], or whether they are facultative/obligate [3], as well as by host biology, and the surrounding biotic or abiotic environment [4]. While hosts can be genetically predisposed to susceptibility [5], disease outcomes can be made worse if hosts have a comorbidity [6] or an impaired or over-reactive immune response [7]. When invading a host, pathogens will also interact with other microbial species [8]. The outcome of infection is thus held in the balance by the complex interactions between a host, its microbiota, and both the biotic and abiotic environment [4].

Microbiota are vital to the functioning of their multicellular host organisms. This realisation has fuelled great interest in the effects of microbes on plant [9] and animal host health [10]. Microbe-mediated protection against infection is a widespread phenomenon across host species [11], with components of the microbiota and their interactions with a host and the wider microbial community mediating susceptibility to invading pathogens and internal pathobionts [12,13]. There are several ways to categorise and define pathogens based on their biology [3]. Here, we use the term “pathobiont” to mean normally harmless components of the microbiota which have pathogenic potential in some contexts [14–16]. We distinguish these from “invading pathogens,” by which we mean pathogens (including parasites) acquired from a source external to the host (i.e., from a different host or from the environment).

It is well established that host microbiota generally play a beneficial role in preventing or fighting infection [17–20]. Microbe-mediated protection can be mediated via resource competition [21,22], interference competition [23], or the host immune response [24,25]. However, the relative magnitude of these benefits might decrease when microbiota components, in some cases, directly or indirectly facilitate the onset of disease caused by invading pathogens or pathobionts (Table 1). Although some invading pathogens can exploit cues or changes in the resource/immunological environment shaped by the microbiota itself, the context of host health is also an important determinant of infection. Diminished host health can remove the beneficial services the microbiota normally provides. Changes in host health can correlate with dysbiosis of host microbiota [26–28], and opportunistic microbiota components can transition to become harmful among the perturbation [26,29–31]. This perturbation and transition of commensals towards pathogenicity can sometimes even be caused by invading pathogens [32,33]. Moreover, protective microbes can become relatively costly to their host in the absence of the invading pathogens they would otherwise suppress [31,34,35] (i.e., the same microbial species is protective in one context, but costly in another; see “Costly protective symbionts”).

**Table 1:** Summary of the drivers and mechanisms by which the microbiota facilitate harmful infection.

Pathway to pathogenesis	Driver	Mechanism	Due to change in host health?	Illustrative example	Other relevant references
Facilitate pathogenic invaders	Niche exploitation	Invading pathogen cross-feeds off microbiota metabolites	No	Human microbiota metabolites increase severity of *Escherichia coli* infection [29]	[39–43]
		Invading pathogen exploits host transmission of microbiota components	No	Trypanosomatid parasite *Leptomonas pyrrhocoris* exploits host transmission of mutualist Coriobacteriaceae microbial symbionts between firebug hosts to aid its own transmission [44]	[45,46]
	Provide cues	Pathogens require contact with microbiota to initiate infection	No	Bacterial surface structures (Type 1 fimbriae) bind to proteins at the poles of *Trichuris muris* worms’ eggs and trigger hatching [47]	[48]
	Alter immunological environment	Microbiota components increase activity of specific immune cells, enhancing susceptibility to infection	No	*Lactobacillis* bacteria in mouse microbiome elevates regulatory T-cell frequencies known to result in greater helminth establishment [49]	
	Lower ecological resistance	Lower microbiota diversity reduces colonisation resistance/competitive exclusion	Yes	Loss of specific microbiota components correlate with onset of *Clostridioides difficile* infection in a mouse model [50]	[51–53]
Facilitate infection from within	Transitions from (low abundance) commensal to (high abundance) pathobiont	Lower microbiota diversity from biotic or abiotic stress to hosts	Yes	Stress in the brook charr fish *Salvelinus fontinalis* induces microbiota dysbiosis, causing reduction in beneficial bacteria and increase in opportunists [28]	[36,54–56]
		Metabolic changes in pathobionts	No	Bacterial nucleoside catabolism of gut luminal uridine to uracil and ribose facilitates the commensal-to-pathogen transition in *Drosophila* microbiota components [57]	[58–60]
		Pathobiont takes advantage of disruption to host homeostasis	Yes	High fat diet and subsequent inflammation in the human gut leads to increase in opportunism from within the microbiota [26]	[61–65]
		Overexpansion of resident pathobiont	Sometimes	Resident *Staphylococcus aureus* overexpansion on the skin corresponds to onset of atopic dermatitis in humans [66]	[67–70]
		Antibiotic treatment	Yes	Antibiotic-mediated alteration of the gut microbiota changes the metabolic profile of this environment to one that favours expansion of *C*. *difficile* in a mouse model [71]	[72–75]
	Within-host translocation	Disruption to gut barrier function and/or bacterial overgrowth	Yes*	In *Manduca sexta* (tobacco hornworm), disruption of the gut epithelial barrier led to translocation of the gut microbiota component *Enterococcus faecalis*, ultimately leading to sepsis [76]	[32,33,77–79]

Illustrative examples for each mechanism and other relevant references provided. We highlight whether a change in host health affects the pathogenic potential of the microbiota.

* Infection by invading pathogens can cause this disruption to host health.

To understand the multifaceted contributors to infectious disease, the potentially harmful aspects of the microbiota and its components warrant consideration. Microbe-based therapies for disease are being investigated as alternatives to antimicrobials for a wide range of animal hosts, from endangered amphibians to humans [36–38]. A thorough evaluation of the potential for host microbiota to contribute to infectious disease is necessary to establish their utility in disease management as anti-infective prophylactics, probiotics, and prebiotics. In this review, we will discuss the conditions under which microbiota can promote or worsen infection outcomes, with evolutionary consequences. We will then discuss the implications of this potential to facilitate pathogen invasion and infection from within for microbiota manipulation.

## Promotion of pathogen invaders

### Microbiota components modify the within-host environment

#### Metabolic environment

Microbiota metabolites are beneficial to hosts in myriad ways. They help to prime the immune system, act as antimicrobials to combat infection, and aid host metabolism [24,80–82]. However, microbiota metabolites can also provide a convenient and easily attainable source of food for invading pathogens to exploit. Metabolic cross-feeding, in which a product of metabolism from one strain is used by another strain, generates novel niches that may benefit pathogens [83]. This assimilation of resources can enhance energy production within the pathogen, enabling increased virulence and rapid growth, and thus more severe disease. For example, the human gut commensal *Bacteroides thetaiotaomicron* (*Bt*) can exacerbate infection caused by enterohaemorrhagic *Escherichia coli* (EHEC) via metabolic cross-feeding [84]. *Bt* modifies the metabolic environment at the site of EHEC infection, increasing metabolites involved in gluconeogenesis which are then sensed by the virulence-regulating transcription factor Cra. Virulence is up-regulated as a result and, concurrent with invasion of the gut epithelial barrier (also facilitated by *Bt*), EHEC induces a greater degree of host pathology and higher risk of mortality.

Individual species of the microbiota cannot always be pinpointed for their role in facilitating infection. While *Bt* was specifically identified in the previous example as a contributor to EHEC infection [84], microbial metabolites from multiple components of the microbiota can also collectively enhance EHEC virulence [85]. A comparison between human and mouse microbiota metabolites illustrated that the increased severity of EHEC infection in humans, compared to that in mice, is driven by distinct human gut microbiota metabolites [29]. These metabolites specifically induce increased expression of flagellin in the pathogen, increasing its ability to invade host tissues. Distinct microbial communities can thus shape different infection outcomes via metabolite production.

The metabolic environment within a host is a crucial contributor to the pathogenesis of invading organisms. It can be extensively modified by components of the microbiota to both the detriment and the benefit of the host. Changes in host health can likewise alter the within-host metabolic environment, contributing to disease onset from resident commensals [62]. Given the diversity of species housed by the animal gut, there are complex interactions to pick apart. Research is moving towards characterising the functionality of the microbiome by holistically sampling its taxonomic and genomic repertoire in addition to the chemical phenotype. Progress has been made in uncovering pathogen-induced disease phenotypes that are enhanced by the microbiota through application of multi-omics strategies [86,87] (see Table 2). Nonetheless, data integration and interpreting meaningful biological signatures of infection (e.g., biomarkers of infection) remain a challenge [88].

**Table 2:** Representative examples of omics approaches used to deduce the role of microbiota components in facilitating infection and worsening infection outcomes.

Approach	Description	Example findings
Proteomics	Characterises the protein profile of community being studied. Potential use in identifying biomarkers of infection within the microbiome.	The saliva proteome of human hosts was found to reflect the dynamics of the oral microbiome, including community changes that lead to disease. Identification of biomarkers within the saliva proteome could be used to diagnose oral infections [97].
Metabolomics	Elucidates specific metabolites present under study conditions. Gives insight into metabolites required for pathogenesis/mutualism by microbiota components.	Antibiotic-mediated alteration of the human gut microbiota shifts the global metabolic profile in this niche towards one that favours *C*. *difficile* infection. Specific metabolites were identified that change in abundance following antibiotic treatment. These changes in tandem benefit *C*. *difficile* [71].
Transcriptomics (also referred to as functional gene expression)	Enables characterisation of the abundance of RNA (transcriptional activity) of both coding and noncoding regions of the genome. This approach is more informative than gene presence/absence.	Differential transcript expression identified in amphibian host populations with different disease history relating to ranavirus infection. Provides information about how hosts respond to infection [98].
Genome-scale metabolic modelling	Integrates genomic information with metabolomics data to create predictive models of metabolism in a given study condition.	Identification of nutrient conditions in a multispecies biofilm model of the human gut that results in *C*. *difficile*–associated dysbiosis. Statistical modelling predicted the experimentally observed metabolic changes causative of an increase in *C*. *difficile* abundance and the subsequent decrease in abundance of protective microbiota components [99].

#### Immunological environment

Microbiota can prime the host immune response, altering their susceptibility to invading pathogens. Pathogen infectivity can be indirectly reduced by host microbiota this way [89–93]. Conversely, launching the immune response can inadvertently boost infection by some infectious agents [49]. Reynolds and colleagues [49] found that Lactobacillaceae species abundance in the mouse duodenum positively correlated with susceptibility to the nematode parasite *Heligmosomoides polygyrus* and heightened immunosuppressive regulatory T-cell and Th17 responses. Subsequent treatment of mice with *Lactobacillus taiwanensis*—a rodent commensal dominant in infected mice—elevated regulatory T-cell frequencies and promoted the establishment of *H*. *polygyrus*. The fact that microbiota composition changed after *H*. *polygyrus* exposure towards more “helpful” bacterial species suggests that parasites could actively modify the microbiota to improve their survival. This manipulation could occur directly via antimicrobials [94] or by pathogen-induced host inflammation [95]. Physical disruption of the host site might also cause changes in resource availability, shifting microbiota composition [96].

### Invading pathogens might evolve in response to host microbiota

Microbes can evolve quickly [100] because of their large population sizes and rapid generation times. Microbiota components can evolve within their host’s lifetime with consequences for host health [101]. For example, a mildly pathogenic strain of the gut microbiota component *Enterococcus faecalis* has been shown in nematode hosts to evolve to become more protective due to competitive interactions with a virulent pathogen [23]. Likewise, the pathogen *Candida albicans* was shown to evolve towards protective mutualism when introduced to a new host in a mouse model [102].

Invading pathogens, in turn, may evolve to overcome or exploit the host microbiota. They can readily overcome barriers to their establishment, including from host resistance [103], antibiotic treatments [104], and vaccines [105]. Theory has shown that pathogens can evolve virulence factors to overcome commensals in the host microbiota, either directly killing their competitors [106] or inducing host inflammation as a form of “proactive invasion” [95]. Experimental evolution approaches in animal model systems have produced mixed evidence on the ability of evolving pathogens to escape suppression by protective microbes. Martinez and colleagues [107] found that niche blocking by *Wolbachia* in *Drosophila melanogaster* effectively suppressed the pathogen *Drosophila* C virus (DCV), which did not evolve to overcome the protective symbiont. In contrast, Rouchet and Vorburger [108] found the parasitoid wasp, *Lysiphlebus fabarum*, readily counteradapted to the protection given by sympatric *Hamiltonella defensa* in aphids. A variety of pathogens may have evolved to exploit host microbiota for replication and transmission. Poliovirus and *Trichuris muris*, for example, have been empirically found to depend on interactions with mouse intestinal microbiota to trigger replication and hatching, respectively, at key host sites [47,48]. Poliovirus was able to better associate with host cells, and its replication was enhanced by up to 500% after binding lipopolysaccharide on enterobacterial surfaces [48]. Similarly, fimbriae on the surface of gut colonisers *E*. *coli* and *Salmonella typhimurium* were found to bind to proteins at the poles of eggs of the parasitic nematode, *T*. *muris*. This interaction with enterobacteria provides an essential cue, triggering the emergence of infective larvae [47].

Microbiome-mediated protection can drive the evolution of increased [109] and decreased [110] pathogen virulence. McNally and colleagues [109] found that manipulating the microbiota generated increased competition between commensal competitors and increased the intensity of bacterial warfare. Using theory, they found that stronger competition selected for increased expression of pathogen weapons (virulence factors). Enhanced production of virulence factors by many pathogenic bacteria can inadvertently harm the host. For example, release of Shiga toxin-encoding phage by shigatoxinagenic *E*. *coli* [111], and similarly TcdA released by *Clostridioides difficile*, can clear commensals both directly and via provocation of host inflammation [112,113].

Host microbiota has the potential to influence the evolutionary trajectory of invading pathogens. Manipulating host microbiota offers a promising route to treat or prevent infection, but such approaches should be scrutinised in light of the evolutionary potential of target pathogens.

### Harmful infection from within

#### Transitions of commensal microbes to pathogens

Commensals in the microbiota can transition along the parasite–mutualist continuum [66,76,114]. Transitions towards pathogenicity can be influenced by changes to the within-host environment—onset of illness or compromised immunity [7], diet [26], antibiotic treatment [115], or stress [28,116]—as well as changes in the external environment [28]. Infection by invading pathogens can also induce otherwise commensal bacteria to become pathogenic [33,117].

A well-studied example of a transition to pathogenicity is that of *C*. *difficile*, the causative agent of colitis. *C*. *difficile* can be at very low abundance in the human gastrointestinal tract. A healthy gut microbiota usually provides colonisation resistance against *C*. *difficile* expansion [52]. However, following a period of antibiotic treatment which diminishes the protective power of the microbiota, this bacterium can proliferate extensively to dominate the intestinal niche [71]. In this context, it is a highly problematic pathogen which can cause recurrent disease. Faecal microbiota transplants have proven useful in such cases, whereby the dysbiotic gut microbiota of a *C*. *difficile* patient is replaced with that of a healthy donor to eliminate the infection [118].

How can these transitions to pathogenicity occur among pathobionts? Metabolic changes in components of the microbiota can underpin the transition. Recent work on the *Drosophila* gut microbiome demonstrates that catabolism of host gut luminal uridine by pathobionts drives the generation of uracil and ribose. These metabolites respectively trigger an inflammatory host immune response and increased expression of virulence genes in pathobionts. Quorum sensing regulates both processes and is therefore necessary for a transition to virulence. Deletion of genes involved in nucleotide metabolism in strains of enteric *Drosophila* pathobionts blocked quorum sensing and thus the commensal-to-pathogen transition. Metabolites such as uracil and ribose may therefore act as pathogen-specific indicators, used by metazoan hosts to distinguish good from bad within the gut. Recognition of these indicators equips hosts to modulate immunity and gut-microbe homeostasis in response to changes within the microbiota [58].

In polymicrobial infections, metabolic cross-feeding can be an essential source of nutrients, enhancing the ability of commensal microbes to establish infection. The pathobiont *Aggregatibacter actinomycetemcomitans*, for example, requires L-lactate produced by the commensal bacterium *Streptococcus gordonii* to establish polymicrobial periodontal infection in a murine abscess model [119]. *A*. *actinomycetemcomitans* also exhibits enhanced respiratory metabolism in the presence of *S*. *gordonii* [120], as the latter increases the bioavailability of oxygen to the opportunist by providing electron acceptors. *A*. *actinomycetemcomitans* uses these electron acceptors to increase energy yield in the form of ATP production, which promotes increased virulence. With more energy available, the pathobiont can invest in the production of toxins, adhesins, and immunomodulatory proteins, among many other virulence factors [120].

Pathobionts have an array of tools available to adapt to environmental change within their niche [69,121–123]. Factors which contribute to the commensal bacterial lifestyle can be repurposed upon immune compromisation in the host or upon nutrient limitation or community disruption of the microbiota. Such changes within the host environment can lead to pathobionts proliferating beyond their niche to invade host tissues [69,123]. Adhesive proteins, for example, are required for asymptomatic colonisation of a new host, yet are also important in attaching to host cells to initiate invasion [123,124]. They can additionally contribute to the development of bacterial biofilms [69,70] to facilitate persistence of an infection under adverse conditions (e.g., antibiotic treatment). Likewise, toxins play a significant destructive role in the onset of disease. Toxins induce host cell lysis and stimulate inflammation, and they are recognised as major drivers of the symptoms of bacterial infection [125]. Recent research has also highlighted the contribution of toxins to pathobiont colonisation or persistence in different niches within the host during asymptomatic carriage, thus they aid both the commensal and pathogenic lifestyles of pathobionts [126]. Gene expression changes underpin transitions to pathogenicity and are driven by the need to adapt to changing conditions [121,122]. Infection can therefore be instigated by pathobionts within the host microbiota, following a transition from commensalism to a pathogenic state.

#### Costly protective symbionts

In wild animal systems, beneficial microbiota components otherwise known as defensive/protective symbionts have been shown to prevent pathogen establishment and reproduction [127]. They are so effective at defending that the evolution of host resistance is slowed in the face of pathogen infection [128]. Many of these symbionts can, however, impose a physiological burden upon their host that is measured in the absence of an invading threat. [127]. For example, while the endosymbiont *Wolbachia* in numerous arthropod hosts defends against parasitic viruses [129], bacteria [130], and nematodes [131], *Wolbachia* in *Drosophila* fruit flies can cause a reduction in colonised host fertility, fecundity, and egg hatch rates, mediated by high symbiont densities [31]. A trade-off emerges in many host-microbe systems whereby increased conferred protection means the symbiont can become more pathogenic [34,35] (albeit, see Cayetano and colleagues [132]). Mathé-Hubert and colleagues [133] further showed that the cost of carrying a protective symbiont (*Spiroplasma*) in pea aphids can be alleviated by concurrent colonisation with a second symbiont (*Regiella insecticola*), as co-colonisation improves host lifetime reproduction and population growth.

Changes in the abiotic environment can also reveal the costs of these resident protectors in the microbiota. One extreme example is a species of the nematode-infecting bacterium *Leucobacter*, which under dry laboratory conditions is a protective bacterium against another highly virulent *Leucobacter* species, but in aqueous conditions causes hosts to become irreversibly fused by their tails leading to death [134]. The abiotic environment can therefore mediate host-associated microbe function to both favour and oppose pathogenicity.

### Microbiota community structure as an early warning signal

Healthy microbiota community compositions can differ between individuals and population groups and also within individuals over time [135]. It is consequently not always feasible to establish what a “typical” dysbiotic microbiota looks like during infectious disease. However, a recent study in apiculture has demonstrated how early microbiota perturbations can have sustained negative consequences on host development and increase pathogen susceptibility within a population [116]. Schwarz and colleagues administered the commensal species *Snodgrassella alvi* to newly emerged worker bees as a potential probiotic therapy to protect against the parasite *Lotmaria passim*. Yet, despite *S*. *alvi* being part of the usual core microbiota of bees, inoculation of this species alone in young hosts led to microbiota perturbation, possibly reducing the protective benefits normally conferred and ultimately increasing parasite susceptibility [116].

While microbiota dysbiosis in general may correlate with infectious disease onset, microbial taxonomic signatures for specific infections may not always be a reliable indicator of disease [136]. The Anna Karenina principle [137] (“all happy families look alike, but each unhappy family is unhappy in its own way”) has been applied to explain observations in which microbiota community composition varies more between diseased individuals than healthy individuals. Nonetheless, in some instances, pathologies may be predicted by a specific reduction in certain key taxa. Bacterial vaginosis (BV) in humans is one such example, a condition caused by dysbiosis within the vaginal microbiota that affects approximately one-third of reproductive age women [138]. Vaginal microbiota composition varies across demographics [139], but onset of BV is typically associated with a reduction in *Lactobacillus* species, accompanied by the dominance of anaerobes and increased alpha diversity [140]. In these lactobacilli-depleted communities, the presence of biogenic amines can increase [141]. These amines, and the microbial community composition with which they are associated, could be useful biomarkers of disease in the early stages of BV development. Indeed, multi-omic approaches have been used to characterise the metabolic profiles corresponding to different symptomatic BV types [142]. Yeoman and colleagues [142] took this approach and identified distinct microbial taxa and metabolites which correlated to 2 different symptomatic BV types (and also to host behaviour). The characteristic odour of BV infection was linked to *Dialister* spp., the presence of discharge was linked with *Mobiluncus* spp., and *Gardnerella* spp. were linked with the symptom of pain. These findings provide both potential diagnostic markers for the onset of disease and insights into the determinants of BV.

Moving beyond correlative relationships between microbes and infections to establishing causation remains a major challenge [143–146]. Due to the complexities of microbial communities within a host, including the high species richness within a niche and the multitude of microbe–microbe and host–microbe interactions, it is often difficult to attribute specific microbes to a causative role in disease. Furthermore, in some cases, infection may not be attributable to one species, but to polymicrobial interactions which are difficult to pick apart [30]. Host heterogeneity in genotype, lifestyle, and diet further compounds the ability to infer causality. Not all components of the microbiota are culturable in the laboratory setting and are only identifiable as members of the community through sequencing. They are thus often excluded from culture-dependent laboratory experiments aiming to determine causality [86,147–150].

To bridge this gap between correlation and causation in elucidating the relationship between microbiota and infection, current research is benefitting from combining laboratory experiments with multidisciplinary and multi-omic approaches (see Table 2). Tractable, controlled experimental models of defined microbial communities will be important in this transition [151]. Synthetic microbial communities composed of native microbiota components are now being developed for use in model organisms [147,152–154]. Such resources will allow in-depth dissection of host–microbiota interactions in model organisms, using tools which are easily controlled while remaining representative of natural systems. The combination of experimental models with corresponding omics data will further allow functional verification of bacterial phenotypes within the microbiota [155]; this mechanistic insight will be essential in determining causality in microbial infections.

### Microbiota manipulation: Always a silver bullet?

Microbial approaches to managing disease in both humans and animals are gaining traction. The application of protective microbes directly to a host, or into a host’s habitat or food source, has been investigated for the control of infectious disease in endangered amphibians [36], aquaculture [156], and apiculture [157] as well as in the prevention and treatment of infectious and noninfectious human disease [38].

Microbe-based solutions have huge potential as alternatives to synthetic drugs [156,158,159]. However, they can sometimes have off-target effects. Studies on amphibian infection reveal the need for identification of these effects associated with probiotic use. Inhibition of the amphibian fungal pathogen *Batrachochtyrium dendrobatidis* (*Bd*) by bacteria can differ based on pathogen genotype and microbial community composition [160,161]. Single bacterial strains show both growth inhibition or promotion depending on *Bd* genotype. Becker and colleagues [37] exposed the critically endangered Panamanian golden frog, *Atelopus zeteki*, to fungal *Bd* and candidate probiotic bacteria identified based on their *Bd* inhibitory activity in vitro. Results of the in vivo study showed no difference in *Bd-*induced mortality in probiotic-treated versus untreated groups. Several probiotics, however, showed a (nonsignificant) trend towards exacerbating *Bd*-induced mortality when compared to *Bd* alone. More recently, a probiotic treatment for the emerging fungal pathogen of amphibians *Batrachochytrium salamandrivorans* (*Bsal*) was shown to slow disease progression, but did not improve individual survival within populations [36]. A longer period of infection resulting from treatment was suggested to likely extend the shedding period of *Bsal* into the environment, increasing its transmission. Research has also shown that colonisation resistance of the native skin microbiota can be metabolically costly and cause amphibians to lose body mass during probiotic treatment for chytridiomycosis [162]. These amphibian studies demonstrate the difficulty in applying protective microbes in the natural environment. There could be a mismatch between in vitro and in vivo outcomes, genetic variation in the effectiveness of protective microbes, or probiotic treatment could alter the infection dynamics in a way that benefits transmission.

Transplantation of entire microbial communities has shown promise in treating human disease. Faecal microbiota transplants are currently used to successfully treat recurrent *C*. *difficile* infection [118]. However, the long-term and off-target effects of this intervention remain unknown [158]. One potential side effect is the unintentional transfer of pathobionts from donor to recipient [163], for which follow-up studies are lacking [164]. Evidence is also emerging of extra-intestinal and systemic effects of intestinal microbiota replacement [165], including obesity [166], autoimmune disorders [167], and depression [168]. Observations of such varied off-target effects reveal the complex and systemic consequences which microbiota manipulation may have on hosts.

The use of known protective microbes as probiotics also needs to be monitored for unexpected consequences. *Bifidobacterium longum* subsp. *longum* has been investigated for its potential to prevent lethal infection from enteric pathogens. This bacterium is a component of the human gut microbiota which positively modifies the metabolic environment within the gut to inhibit translocation of invading EHEC from the gut to the blood [169]. Severe and ultimately lethal infection is prevented in this manner, but cases of infection caused by this species have been reported [30]. Tena and colleagues [30] reflected *that B*. *longum* may often be overlooked as a cause of disease in polymicrobial infections due to being labelled as a commensal.

Administration of protective microbes used clinically as probiotics could be particularly problematic for immunocompromised, critically ill, or otherwise vulnerable hosts [170]. Safety concerns include the potential for a probiotic to cause infection by translocation [171], to pass antibiotic resistance genes or other virulence-associated genes onto other microbiota components, and the possibility for production of metabolites that can be toxic [172]. There is also the possibility of permanent colonisation [173] and long-term side effects. Such safety concerns will be essential to account for in cases where probiotic treatments are being investigated to treat vulnerable hosts. Furthermore, the applied probiotic will interact with host microbiota and invading pathogens. As probiotics are inherently “live microorganisms” [174], they retain the ability to evolve, and it is largely unclear how they might change in a new host [175].

## Conclusions

The “microbiome revolution” is revealing the interconnectedness between a host’s health and its resident microbial species. Microbiota components can form an effective non-immunological line of defence against infection [11,17–20]. Although the microbiota can aid pathogens, worsen infection outcomes, or become harmful themselves in the situations we describe, overall it is acknowledged that the benefits of microbiota substantially outweigh any costs.

There is a need to distinguish the different conditions under which microbiota might facilitate infection. Some pathogens and pathobionts can directly exploit the metabolic and immunological environment shaped by the host microbiota. Whether these outcomes are specific to the interacting host and pathogen species/genotype is unclear. A change in host health status may dictate whether microbiota have the potential to allow for harmful infection [176,177]. Poor health, the application of antibiotics, or infection by invading pathogens might cause a loss of microbiota diversity (and thus protective traits) or physical disruption to the environment allowing for the expansion of harmful microbes. The integration of bioinformatics with lab experiments in model systems will help to characterise genomic, proteomic, and metabolic features of the microbiome in different contexts of infection [147,152,154,155,178,179]. Functional gene expression studies [180–182] and genome-scale metabolic models are also proving increasingly powerful in characterising the microbiota profiles of healthy versus diseased individuals [183,184]. Overall, these approaches will allow predictions to be made about microbial phenotypes (e.g., metabolic traits, toxin production, and antibiotic resistance) in different contexts and the relevance of these phenotypes to infectious disease.

There are further outstanding questions regarding the contribution of microbiota components to infection in real time. In vitro and in vivo coculture experiments using communities representative of native host microbiota [152] can reveal antagonistic, competitive, and beneficial interactions between species within the microbiota, as well as between microbiota and invading pathogens [185,186]. Interactions between microbiota and host immunity can also be more intricately explored in model animal systems to study the role of the immunological environment in infection promotion [187–189]. With a better understanding of the interactions and dynamic processes that govern the microbiota, it may be possible to predict when harmless components will promote invading pathogens or become pathogenic themselves. Direct experimental tests in tractable systems will help to move our understanding beyond correlations of microbiota structure with infection outcomes and host health.

Thinking on an evolutionary timescale is essential for tackling why pathogens can benefit from the host microbiota. Systems in which pathogenic invaders depend on microbiota to start replicating [47,48] may indicate a coevolutionary relationship in which host-associated microbial species and pathogens cooperate to promote their establishment within the host. The potential for coevolution between protective microbes and pathogens has been demonstrated experimentally [190]. The extent to which pathogen exploitation of microbiome metabolites and immune priming is incidental, or the product of adaptation, remains unclear. Perhaps pathogens can evolve to improve their exploitation of host microbiota. Pathogens might also gain a competitive advantage by modifying their within-host environment (“niche construction” [191]) to select host-associated microbes most favourable to their survival [192]. The long-term effectiveness of a manipulated microbiota will also be vulnerable to pathogen evolution. Does engineering the microbiota or therapeutically applying microbes drive unwanted evolutionary changes in the target pathogen? Reductions in pathogen virulence could be desirable. However, any pathogen adaptation and increased within-host fitness might enhance their transmissibility in the host population. Most of our current understanding of the evolutionary biology in this area is based on theory and empirical work in model systems. Its relevance to human infections is an open question.

Microbiota are an important driver of variation in the prevalence and severity of some infections. Pathogen-suppressive forces generally dominate, but the interactions within the microbiota and between microbiota and invading pathogens are complex and need more direct empirical investigation. Nevertheless, shining a light on the potential ways in which the microbiota can sometimes facilitate infection by pathogens or pathobionts is critical for understanding patterns of infection in natural and applied settings.
